# Who Tests, Who Doesn't, and Why? Uptake of Mobile HIV Counseling and Testing in the Kilimanjaro Region of Tanzania

**DOI:** 10.1371/journal.pone.0016488

**Published:** 2011-01-31

**Authors:** Jan Ostermann, Elizabeth A. Reddy, Meghan M. Shorter, Charles Muiruri, Antipas Mtalo, Dafrosa K. Itemba, Bernard Njau, John A. Bartlett, John A. Crump, Nathan M. Thielman

**Affiliations:** 1 Duke Global Health Institute, Duke University, Durham, North Carolina, United States of America; 2 Center for Health Policy, Duke University, Durham, North Carolina, United States of America; 3 Duke University Medical Center, Durham, North Carolina, United States of America; 4 KIWAKKUKI (Women Against AIDS in Kilimanjaro), Moshi, Tanzania; 5 Kilimanjaro Christian Medical Centre, Moshi, Tanzania; 6 Kilimanjaro Christian Medical College, Tumaini University, Moshi, Tanzania; University of Cape Town, South Africa

## Abstract

**Background:**

Optimally, expanded HIV testing programs should reduce barriers to testing while attracting new and high-risk testers. We assessed barriers to testing and HIV risk among clients participating in mobile voluntary counseling and testing (MVCT) campaigns in four rural villages in the Kilimanjaro Region of Tanzania.

**Methods:**

Between December 2007 and April 2008, 878 MVCT participants and 506 randomly selected community residents who did not access MVCT were surveyed. Gender-specific logistic regression models were used to describe differences in socioeconomic characteristics, HIV exposure risk, testing histories, HIV related stigma, and attitudes toward testing between MVCT participants and community residents who did not access MVCT. Gender-specific logistic regression models were used to describe differences in socioeconomic characteristics, HIV exposure risk, testing histories, HIV related stigma, and attitudes toward testing, between the two groups.

**Results:**

MVCT clients reported greater HIV exposure risk (OR 1.20 [1.04 to 1.38] for males; OR 1.11 [1.03 to 1.19] for females). Female MVCT clients were more likely to report low household expenditures (OR 1.47 [1.04 to 2.05]), male clients reported higher rates of unstable income sources (OR 1.99 [1.22 to 3.24]). First-time testers were more likely than non-testers to cite distance to testing sites as a reason for not having previously tested (OR 2.17 [1.05 to 4.48] for males; OR 5.95 [2.85 to 12.45] for females). HIV-related stigma, fears of testing or test disclosure, and not being able to leave work were strongly associated with non-participation in MVCT (ORs from 0.11 to 0.84).

**Conclusions:**

MVCT attracted clients with increased exposure risk and fewer economic resources; HIV related stigma and testing-related fears remained barriers to testing. MVCT did not disproportionately attract either first-time or frequent repeat testers. Educational campaigns to reduce stigma and fears of testing could improve the effectiveness of MVCT in attracting new and high-risk populations.

## Introduction

Universal testing and immediate treatment of HIV-infected individuals could dramatically reduce or even eliminate HIV transmission [1], [2]. Yet, despite global expansion of HIV counseling and testing options [3], serostatus awareness is low in heavily affected communities in sub-Saharan Africa [4], [5], and late presentation to care remains common [6]. There is a pressing need to understand which HIV counseling and testing strategies are most successful in recruiting new and high risk testers in order to improve testing uptake and facilitate early linkage to care.

Mobile HIV voluntary counseling and testing (MVCT) has been promoted as a means of reaching populations with limited access to HIV testing, and has been effective in attracting large numbers of new testers in countries such as Tanzania, Zimbabwe and Cameroon [7], [8], [9], but there are limited data describing the characteristics of community members who do not access this opportunity. While early data from a large randomized study offering in-village mobile testing demonstrated 3–13 times greater voluntary counseling and testing (VCT) uptake in intervention villages compared with control villages which had access only to nearby facility-based VCT, only 30% of residents ages 18–32 had accessed the intervention in the Tanzania sites after 24 months [4]. In 2007 and 2008, a national voluntary counseling and HIV testing campaign in Tanzania, which included a substantial MVCT component, attracted more than 4.8 million testers [9]. However, no information is available about the extent to which new or high risk clients presented for testing, and it remains unknown whether such campaigns, particularly in rural areas, can overcome important non-economic barriers to HIV testing, including HIV related stigma and fears of testing [10], [11]. Whether expanded HIV testing strategies reduce such barriers and successfully attract new and high risk testers, or preferentially attract lower risk repeat testers, has substantial implications for the strategies' cost effectiveness and for the possible success of universal testing and treatment policies.

Comparing characteristics of MVCT participants and randomly selected community residents who did not participate in MVCT, we examined selection effects, which we defined as systematic differences between MVCT participants and community residents who did not undergo VCT, in MVCT campaigns across four rural village clusters in the Kilimanjaro Region of Tanzania. We evaluated differences in socioeconomic and HIV risk characteristics, HIV testing histories, HIV related stigma, and attitudes toward testing, to assess the extent to which MVCT campaigns attracted new and high risk testers and reduced barriers to HIV testing.

## Methods

### Ethics Statement

Written informed consent was obtained from all study participants prior to enrollment. Ethical approval was obtained from the Kilimanjaro Christian Medical College Research Ethics Committee, a Duke University Health System Institutional Review Board, and the Tanzanian National Institute of Medical Research National Medical Research Coordinating Committee.

### Study Design

The study was conducted between December 2007 and April 2008 in 4 rural village clusters in three districts of the Kilimanjaro Region of Tanzania. In each village, randomly selected community respondents aged 18 to 50 years were given HIV Awareness, Attitudes, and Risk Surveys (HAARS). Subsequently, free MVCT was offered, and HAARS were administered to eligible clients during pretest counseling. HIV risk, testing history, socioeconomic characteristics, and HIV-related stigma were compared between MVCT participants and randomly selected community respondents who did not access MVCT.

#### Village Selection

Villages were chosen on the advice of local leaders, and priority was given to communities that did not have in-village HIV testing services at the time of project planning. Population sizes of the village clusters ranged from 2,600–4,500, with a total population of 15,400, of whom 6,300 (41%) were estimated to be between the ages of 18 to 50 years [12]. The distance from villages to the nearest towns or urban centers was approximately 10 to 15 kilometers.

#### Community Sample

Aerial photographs were used to define the geographic sampling frame and to randomly select 150 index structures or buildings in each village cluster. In each structure, one male and one female respondent were randomly selected from all male and female residents aged 18–50. Replacement structures were selected when no eligible respondent resided in the structure or no eligible respondent was enrolled after up to three contact attempts.

### Mobile VCT program and clients

Following the HAARS assessments, free MVCT was offered for 2 to 3 weeks in up to 2 locations per village. Testing locations typically included dispensaries, ward offices, and schools. MVCT staff were employed by KIWAKKUKI, a women-led HIV/AIDS service organization in nearby Moshi, Tanzania; none of the team members were residents of any of the four study villages. Campaigns were advertised at ward leaders' offices, churches, and other key places, as well as via bullhorn advertising on the 2 days preceding and the first day of testing at each location. Consenting MVCT clients aged 18 to 50 years completed pretest counseling and a HAARS assessment. Phlebotomy and rapid HIV-testing were followed by post-test counseling, according to Tanzanian Ministry of Health Guidelines [13]. Samples were tested using Capillus (Trinity Biotech PLC, Bray, County Wicklow, Ireland) and Determine (Abbott Laboratories, Abbott Park, IL, U.S.A.) rapid HIV1/2 antibody tests [14]. Blood samples with contradictory test results, and every 20th blood sample (for quality assurance), were sent to the zonal referral hospital for confirmatory testing via Vironostika HIV-1 microElisa assay (Organon Teknika, Charlotte, N.C., U.S.A.).

### HIV Awareness, Attitudes, and Risk Surveys (HAARS)

Trained surveyors and VCT counselors, respectively, administered HAARS in respondents' homes (community sample) and during pre-test counseling at the MVCT sites (MVCT clients). HAARS included demographic characteristics, sexual history and current sexual practice, HIV testing history, HIV knowledge, perceived HIV risk, and HIV-related fears and stigma.

### Study Population

883 MVCT clients and 644 community respondents aged 18 to 50 years participated in HAARS. Nine community respondents (1.4%) and 5 MVCT clients (0.6%) who reported that they had previously tested positive for HIV were excluded from analyses. Of the remaining 635 randomly selected community respondents, 129 (20.3%) subsequently participated in MVCT and were analyzed as MVCT testers; 506 respondents who did not participate in MVCT formed the comparison group.

### Analysis Overview

Factors associated with MVCT participation, risk selection among MVCT clients, and barriers to HIV testing, were evaluated by comparing characteristics of clients presenting for HIV testing and randomly selected community respondents who did not present for testing. Gender-specific multivariable logistic regression models were used to identify correlates of MVCT participation.

### Measures

#### Socio-demographic characteristics

Socio-demographic characteristics included respondent age (18–24; 25–29; 30–39; 40+ years), marital status (married; divorced; widowed; single), and education (any secondary education vs. none), whether the respondent had any children, weekly household expenditures above or below 10,000 Tanzanian Shillings (TZS), and respondents' main source of income (“farming”; “other stable income source” which included business, skilled workers, salaried workers, and students; and “unstable income source” including unskilled workers, the unemployed, and respondents with other income sources).

#### Risk of HIV seropositivity

Participants' risk of HIV seropositivity was described by variables in two domains. Socio-demographic correlates of HIV infection included respondents' age and marital status, unemployment, and whether the respondent had any children. Exposure risk was described by the number of lifetime sexual partners; multiple sexual partners in the past 3 months; whether any partner had ever tested HIV seropositive; whether the respondent suspected that any of their partners had HIV; and whether any of the respondent's sexual partners had died. Risk variables were combined into indices describing clients' socio-demographic risk, exposure risk, and total risk of HIV infection. Contributions of each risk factor to HIV seropositivity were identified using data from a cohort of 5,628 clients presenting for HIV testing between 2005 and 2008 at a freestanding VCT site in nearby Moshi, Tanzania (KIWAKKUKI; see Table S1 for details). Data from this cohort were previously used to assess correlates of HIV infection and trends in rates of HIV seropositivity among VCT clients in the Kilimanjaro Region of Tanzania [15], [16]. Risk indices ranged from 0 (low risk) to 10 (high risk).

#### HIV testing history

The time since participants' most recent HIV test was categorized as within 1 year; 1 to 2 years; and 3 or more years; persons with no prior HIV test comprised the reference group. Clients were also asked about the total number of times they had previously tested for HIV, categorized as 0, 1, 2, 3, and 4 or more tests.

#### HIV related stigma

HIV-related stigma was assessed using 11 questions in two domains, “internal stigma” (6 questions) and “witnessed stigma” (5 questions; see Table S2). Responses in each domain were added to create stigma scores, with higher scores indicating higher degrees of stigma (see Table S2). Stigma indicators were recommended for use in developing countries and previously validated in Tanzania [17].

#### Barriers to HIV testing

Two questions, previously used by NIMH Project Accept [18], [19], assessed fears of HIV testing and test disclosure: “Most people in my area who want to get tested for HIV are afraid to get tested;” and “Most people in my area who want to get tested for HIV don't want other people to find out if they get tested.” Response options were “strongly disagree”, “disagree”, “agree”, and “strongly agree;” responses were coded from 0 to 3. Clients who had not previously tested for HIV were also asked about reasons for not having tested, including the cost of the test or transportation, distance to testing sites, inability to leave work, doubts about the confidentiality of the test result, and other reasons.

### Statistical Analysis

Gender-specific logistic regression models predicting MVCT participation were used to compare characteristics of MVCT clients and non-participating community clients. Bivariable and multivariable logistic regression models evaluated associations of MVCT participation with socio-demographic, economic, and HIV risk characteristics, as well as HIV testing histories, HIV related stigma, and testing-related fears. Among respondents who reported no prior HIV test, multivariable models assessed associations of MVCT participation with HIV risk and reasons for never having been tested. Differential distributions of reasons for never having previously tested between first-time testers and community-based never testers were used to draw inferences about barriers to testing. Models were estimated with robust standard errors and village level fixed effects.

#### Weighting

The MVCT cohort was comprised of all eligible testers in the four study villages. However, the cohort of non-testers represented only a random sample of eligible non-testers. To allow for estimates of the effects of differences between testers and non-testers on *rates* of MVCT participation, multivariable regression models were estimated with sampling weights to account for eligible community members not included in either the MVCT or community cohorts. Sampling weights were defined as village and gender-specific ratios of MVCT clients to randomly selected community respondents who presented for testing. Estimates from weighted regression models were used to simulate the effects of correlates of MVCT participation on rates of testing in the four study villages.

## Results

### Characteristics of study participants

Of 680 eligible residents in randomly selected households, 644 (94.7%) agreed to participate; 397 (61.6%) of these were female; 341 (53.0%) had previously tested for HIV. Nine (1.4%) reported being HIV-infected. MVCT was attended by 917 clients aged 18–50 years; 883 (96.3%) agreed to participate in the study (ranging from 146 to 289 clients per village). MVCT participants comprised 13.9% of the estimated 6,300 age-eligible residents. Of participating MVCT clients, 423 (47.9%) were female; 432 (48.9%) had previously tested for HIV. 129 MVCT clients (14.6%) were part of the randomly selected community sample (range across villages: 9.8% to 20.6%, p = 0.007). Thirty-five MVCT clients (3.9%) tested HIV seropositive; five (14.3%) of these clients reported that they were previously aware of their infection and were excluded from subsequent analyses. Rates of HIV seropositivity among the remaining 878 MVCT clients ranged from 1.4 to 4.5% across villages (p = 0.323).

### Socio-demographic characteristics

In bivariable comparisons, there were few differences in socio-demographic characteristics between MVCT clients and community respondents who did not present for testing (Table 1). Testers were more likely to be between the ages of 18–24; male clients were more likely to report “unstable” income sources, and less likely to have any children. Female clients were more likely to be divorced, to be unemployed, to report weekly household expenditures below 10,000 TZS, and to have had a health care visit in the past year. Results were similar for all clients and clients who reported no previous HIV test (not shown). Combining age, marital status, unemployment and children, male MVCT clients had a lower socio-demographic risk of HIV seropositivity than non-testers (OR 0.92, 95% CI 0.86 to 0.98); the difference was not significant for females.

**Table 1:** Sociodemographic characteristics of MVCT clients and randomly selected community respondents who did not present for testing: estimates from bivariable logistic regression models predicting MVCT participation.

		MaleTesters	Non-testers	Odds Ratios 4		FemaleTesters	Non-testers	Odds Ratios 4	
		*%*		*OR*	[95% CI]	*%*		*OR*	[95% CI]
*Number of observations*		460	187			418	319		
*Age* 1	18–24	30.4	22.5	*ref*		26.6	23.3	*ref*	
	25–29	16.1	16.0	0.68	[0.39–1.20]	16.5	18.6	0.77	[0.49–1.23]
	30–39	29.3	36.9	0.53**	[0.33–0.86]	27.8	32.4	0.78	[0.52–1.17]
	40–50	24.1	24.6	0.65	[0.39–1.09]	29.2	25.8	1.07	[0.71–1.61]
*Marital status* 1	Married	50.0	52.4	*ref*		64.4	69.2	*ref*	
	Divorced	6.3	4.8	1.45	[0.66–3.18]	10.0	5.3	2.18**	[1.20–3.94]
	Widowed	1.1	0.5	2.19	[0.24–20.03]	5.3	2.8	2.00	[0.89–4.50]
	Single	42.6	42.2	1.13	[0.77–1.64]	20.3	22.6	0.98	[0.68–1.43]
*Secondary education*		18.3	20.9	0.99	[0.63–1.54]	13.4	13.2	1.11	[0.71–1.76]
*Weekly household expenses <TSH 10,000*		23.9	18.2	1.36	[0.87–2.12]	32.5	23.6	1.47*	[1.04–2.05]
*Income source* 2	‘Stable’ income	44.7	47.6	*ref*		51.6	51.7	*ref*	
	Income from farming	28.3	38.5	0.80	[0.52–1.22]	28.1	31.5	0.81	[0.57–1.16]
	‘Unstable’ income	27.0	13.9	1.99**	[1.22–3.24]	20.4	16.7	1.05	[0.69–1.60]
*Unemployed* 1		1.1	2.1	0.36	[0.09–1.40]	1.2	11.9	0.07**	[0.03–0.20]
*Any children* 1		59.8	69.0	0.61*	[0.41–0.90]	85.2	88.7	0.72	[0.46–1.15]
*Health care visit in the past year*		51.1	47.1	1.23	[0.87–1.72]	64.1	51.6	1.71**	[1.24–2.35]
*Socio-demographic HIV risk index (0- 10)* 3		3.9	4.4	0.92*	[0.86–0.98]	3.8	4.0	0.95	[0.88–1.03]

1 Components of the risk index describing sociodemographic correlates of HIV infection;

2 “stable” income includes business, students, skilled, and salaried labor; “unstable” income include unskilled labor, other income, and unemployment;

3 risk index calculated using parameter estimates from Table S1, rescaled to range from 0 (minimum) to 10 (maximum);

4 Bivariable odds ratios and 95% confidence intervals (CI) from logistic regression predicting MVCT participation, controlling for village effects.

### HIV exposure risk

Female MVCT clients were more likely than female non-testers to report a greater number of lifetime sexual partners (OR 2.03, 95% CI 1.28 to 3.22 for 3 lifetime partners, and 2.08, 95% CI 1.22 to 3.55 for 4 to 5 partners; Table 2); differences were not significant for males. Both male and female MVCT clients were more likely than non-testers to report two or more sexual partners in the past 3 months (OR 3.10, 95% CI 1.23 to 7.80 and OR 5.28, 95% CI 1.48 to 18.85, respectively); persons with one sexual partner in the past 3 months were least likely to test. Combining the number of lifetime partners, any sexual partner's HIV infection, and suspicions about partners' HIV infection, MVCT clients exhibited a 21 to 25 percent greater estimated exposure risk than community-based non-testers (OR 1.20, 95% CI 1.04 to 1.38 for males; OR 1.11, 95% CI 1.03 to 1.19 for females).

**Table 2:** HIV exposure risk of MVCT clients and randomly selected community respondents who did not present for testing: estimates from bivariable logistic regression models predicting MVCT participation.

				*Males*				*Females*	
		Testers	Non-testers	Odds Ratios 3		Testers	Non-testers	Odds Ratios 3	
		*%*		*OR*	[95% CI]	*%*		*OR*	[95% CI]
*Number of observations*		460	187			418	319		
*Lifetime partners* 1	0-1	16.8	18.3	*ref*		40.5	49.1	*ref*	
	2	12.9	16.1	0.82	[0.44–1.54]	21.6	24.5	1.16	[0.78–1.73]
	3	17.6	21.5	0.85	[0.47–1.52]	19.7	12.6	2.03**	[1.28–3.22]
	4-5	22.0	19.9	1.11	[0.62–1.99]	13.9	8.8	2.08**	[1.22–3.55]
	6+	30.7	24.2	1.27	[0.71–2.26]	4.3	5.0	1.12	[0.53–2.38]
*Partner in past 3 months*	0	34.1	29.0	*ref*		22.5	17.3	*ref*	
	1	52.8	67.7	0.63*	[0.42–0.94]	70.3	81.8	0.62*	[0.42–0.91]
	2+	13.0	3.2	3.10*	[1.23–7.80]	7.2	0.9	5.26*	[1.48–18.79]
*Any partner tested HIV positive (%)* 1		0.9	0.0	n/a		0.7	0.0	n/a	
*Suspects any partner has HIV (%)* 1		5.0	2.1	2.34	[0.79–6.93]	1.4	0.3	5.67	[0.69–46.57]
*Any sexual partner died (%)*		7.2	7.5	0.90	[0.47–1.71]	7.9	5.0	1.58	[0.86–2.92]
*HIV exposure risk index* *(range 0 to 10)* 2		1.5	1.2	1.20*	[1.04–1.38]	2.7	2.2	1.11**	[1.03–1.19]

1 Components of the risk index describing HIV exposure risk;

2 risk index calculated using parameter estimates from Table S1, rescaled to range from 0 (minimum) to 10 (maximum);

3 bivariable odds ratios from logistic regression predicting MVCT participation, controlling for village fixed effects; n/a indicates that odds ratios and confidence intervals could not be estimated.

### HIV testing history

Controlling for MVCT clients' socio-demographic and exposure risk, there was no significant association between MVCT participation and the time since respondents' last HIV test, though clients whose most recent test was three or more years ago appeared least likely to participate (Table 3). Persons who had previously tested 4 or more times were least likely to test again during the campaigns.

**Table 3:** HIV risk selection and testing history among MVCT clients relative to community based non-testers: estimates from multivariable logistic regression models predicting MVCT participation.

				*Males*				*Females*	
		Testers	Non-testers	Odds Ratios 2,3		Testers	Non-testers	Odds Ratios 2,3	
		*%*		*OR*	[95% CI]	*%*		*OR*	[95% CI]
*Number of observations*		458	186			417	318		
*Risk indices (0 to 10)* 1,2	Exposure risk	3.9	4.4	0.89**	[0.82–0.96]	3.8	4.0	0.92	[0.84–1.01]
	Demographic risk	1.5	1.2	1.26**	[1.08–1.47]	2.7	2.2	1.09*	[1.01–1.19]
	Total risk	3.7	3.9	0.92	[0.84–1.01]	4.4	4.3	1.00	[0.92–1.08]
*Time since last HIV test* 3	Never tested	54.8	59.7	*ref*		46.8	42.6	*ref*	
	Past year	38.4	30.1	1.42	[0.93–2.17]	36.5	35.6	0.76	[0.53–1.09]
	1-2 years ago	3.9	3.2	1.90	[0.75–4.86]	12.2	14.8	0.66	[0.40–1.10]
	3+ years ago	2.8	7.0	0.53	[0.23–1.25]	4.6	6.9	0.58	[0.28–1.20]
*Number of previous tests* 3	None	54.8	59.7	*ref*		46.8	42.6	*ref*	
	1	24.5	14.0	1.88*	[1.10–3.19]	25.7	18.3	1.02	[0.67–1.57]
	2	12.7	11.3	1.33	[0.74–2.41]	18.7	19.6	0.75	[0.49–1.17]
	3	5.2	8.6	0.79	[0.40–1.57]	6.7	14.5	0.38**	[0.21–0.67]
	4 or more	2.8	6.5	0.46	[0.19–1.15]	2.2	5.0	0.30*	[0.12–0.78]

1 Risk indices calculated using parameter estimates from Table S1, rescaled to range from 0 (minimum) to 10 (maximum);

2 odds ratios from multivariable logistic regressions predicting MVCT participation, controlling for time since last HIV test and village effects;

3 odds ratios from multivariable logistic regression models predicting MVCT participation, controlling for estimated total risk and village effects.

Note: 5 clients with missing information on testing history excluded from analyses.

### HIV related stigma and testing-related fears

**Figure 1:**
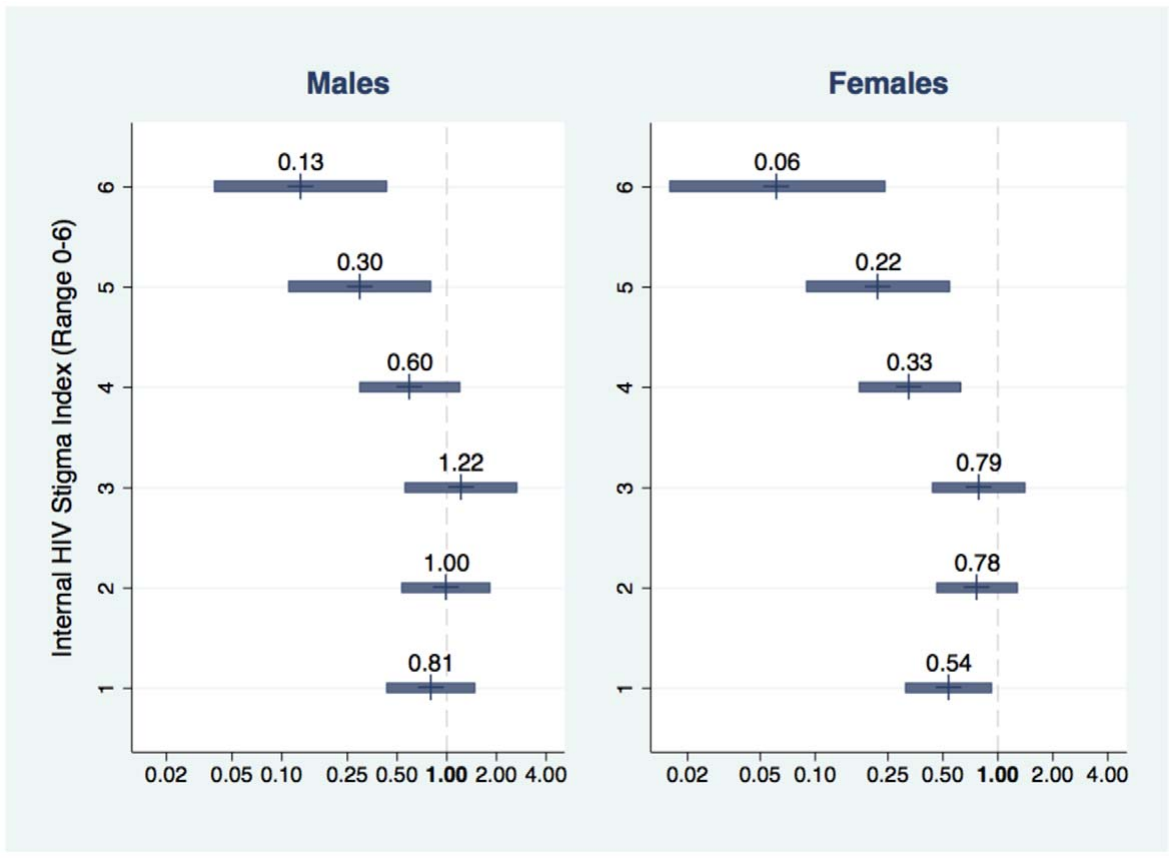
Odds ratios for participation in mobile HIV voluntary counseling and testing by internal HIV stigma index. Odds ratios and 95% confidence intervals are shown for MVCT participation, relative to stigma score of 0. Controlling for HIV risk and testing history, each additional internal stigma item endorsed, on average, was associated with 16% lower odds of MVCT participation for men and 22% lower odds of testing for women.

Controlling for HIV risk and testing history, HIV related stigma and fears of testing or test disclosure were negatively associated with presentation to MVCT (Table 4; Figure 1). Controlling for HIV risk and testing history, each additional internal stigma item endorsed, on average, was associated with 16% lower odds of MVCT participation for men and 22% lower odds of testing for women; higher fears were associated with 28% to 54% lower odds. Compared with persons not endorsing any internal stigma items (17.8%) those endorsing 4 or more items (19.4%) were less than half as likely to participate in MVCT (OR: 0.41, 95% CI: 0.22 to 0.76 for males; OR: 0.25, 95% CI: 0.14 to 0.43 for females; not shown). A simulation using marginal effects estimates from regression models including both stigma and testing related fears suggests that a 50% reduction in HIV related stigma and testing related fears would result in an 80% increase in MVCT participation among men and more than a doubling among women (both p<0.001; not shown).

**Table 4:** Barriers to MVCT participation: estimates from multivariable logistic regression models predicting MVCT participation.

			Males				Females	
	Testers	Non-testers	Odds Ratios^3^		Testers	Non-testers	Odds Ratios^3^	
	Mean or %		OR	[95% CI]	Mean or %		OR	[95% CI]
All persons	458	186			417	318		
HIV Stigma Indices^1^								
Internal stigma (0-6)	2.0	2.4	0.84**	[0.74–0.94]	1.8	2.3	0.78**	[0.71–0.87]
Witnessed stigma (0-5)	0.8	1.0	0.90	[0.77–1.05]	0.9	1.0	1.06	[0.93–1.20]
Attitudes re: HIV testing^2^								
Fear of testing (0-3)	2.1	2.4	0.53**	[0.39–0.71]	2.1	2.5	0.46**	[0.36–0.60]
Fear of test disclosure (0-3)	2.2	2.4	0.72*	[0.54–0.96]	2.1	2.5	0.50**	[0.39–0.64]
Never testers only	251	111			195	135		
Reasons cited for never having tested (%)								
Too expensive to pay for test or transport	9.2	9.9	0.90	[0.36–2.27]	14.4	10.4	1.66	[0.72–3.83]
Testing site too far from home	39.8	22.5	2.17*	[1.05–4.48]	43.1	17.0	5.95**	[2.85–12.45]
Didn't know where to get tested	14.7	4.5	2.17	[0.66–7.11]	20.5	5.9	2.83*	[1.05–7.67]
Can't leave work to get tested	23.9	38.7	0.38**	[0.21–0.71]	7.7	25.2	0.11**	[0.04–0.30]
Doubted confidentiality of test results	3.6	8.1	0.48	[0.14–1.68]	6.7	8.1	0.98	[0.32–2.99]
Other reasons	49.8	71.2	0.48*	[0.26–0.86]	55.4	73.5	0.64	[0.32–1.30]

1 See Table S2 for components of stigma indices;

2 refer to the methods section for definition;

3 models control for clients' estimated risk of HIV seropositivity, time since last test and number of prior tests, if applicable, and village effects.

### Reasons for not having tested previously

Nearly half of the female study participants and more than half of the male participants had never tested for HIV. The most common reasons for never having tested were distance from available testing sites (33%), not being able to leave work (22%), and not knowing where to get tested (13%). First time testers in the MVCT cohort were two to six times as likely as never testers in the community cohort to cite distance to testing places (OR 2.17, 95% CI 1.05 to 4.48 for males; OR 5.95, 95% CI 2.85 to 12.45 for females) and lack of knowledge about available testing options (OR 2.17, 95% CI 0.66 to 7.11 for males; OR 2.83, 95% CI 1.05 to 7.67 for females) as reasons for never having previously tested. By contrast, MVCT clients were significantly less likely to cite not being able to leave work as a reason for not having previously tested. Follow-up assessments among 336 non-testers (67%; results not shown) indicated that 9% were unaware of the MVCT campaigns. Of the remaining respondents 25% disclosed that they were traveling, 20% could not leave work, and 23% mentioned multiple or recent HIV tests as reasons for not participating. Eleven percent were either afraid to receive test results, did not want others in the village to see them testing, or worried about test confidentiality. Six percent had never thought about getting tested. Twenty-two percent cited other reasons for not attending MVCT, such as being busy, forgetting, health issues, or partner disagreement or unavailability.

## Discussion

MVCT campaigns in four villages in rural Kilimanjaro, Tanzania attracted nearly nine hundred testers during 60 days of testing; approximately half were female and half were first-time testers. Relative to community respondents who did not present for testing, MVCT attracted persons with greater HIV exposure risk. MVCT clients reported greater numbers of recent and lifetime partners and higher rates of known or suspected HIV-infected partners. MVCT also appeared to help overcome socioeconomic barriers to testing. Compared with non-testers, female MVCT clients were more likely to report low weekly household expenditures; male clients were more likely to report unstable income sources. First-time testers were significantly more likely than never testers to report distance from testing sites as a reason for never having tested previously. There was no indication that MVCT disproportionately attracted first time or frequent repeat testers.

MVCT, even when conducted by non-residents of the villages, did not appear to overcome HIV related stigma or fears of testing or test disclosure. AIDS-related stigma has been identified in other African settings as an important factor in testing decisions [10], [11], [20], [21], [22]. In Uganda, focus group discussions have highlighted how even home-based testing programs can be limited by stigma and that family-inflicted stigma is a particular concern among women [21]. Among patients with tuberculosis in Ethiopia, qualitative work also suggested that stigma is major reason for non-acceptance of HIV testing [22]. Incorporating community-based educational campaigns aimed at mitigating stigma and fears of testing or test disclosure may improve the effectiveness of MVCT campaigns in attracting additional at-risk clients [4], [23], [24], [25].

Several additional findings of this study deserve mention as they have the potential to inform HIV counseling and testing related policy. First, among women, a recent health care visit was associated with increased odds of presentation for MVCT. While illness may be a common determinant for the utilization of both services, 58% of female and 47% of male MVCT clients who had not previously tested reported a health care visit in the past year, suggesting persisting missed opportunities for provider-based HIV testing.

Second, local accessibility of MVCT did not appear to overcome working persons' barriers to testing. Not being able to leave work was the second most frequently cited reason for never having tested previously; it was also associated with significantly reduced odds of MVCT participation. It may be necessary to expand MVCT availability to evening hours and weekends to allow these persons to get tested, or to promote HIV testing campaigns in the workplace, particularly if such testing can be linked to care [26].

Third, the lack of differences in the overall risk distribution and testing histories between MVCT clients and persons who did not present for testing suggests that rates of seropositivity at MVCT campaigns may be useful proxies for HIV prevalence estimates at local levels. On a large scale, this is supported by national rates of seropositivity during Tanzania's national HIV testing campaign (5.4%) [9], which were comparable to seroprevalence estimates from the 2007-08 Tanzania HIV/AIDS and Malaria Indicator Survey (5.7%) [27]. However, repeat testing of clients who previously tested positive remains a concern: 5 HIV infected MVCT clients (14%) reported to have previously tested positive; self-reported testing histories among other HIV infected clients (3 HIV infected clients reported a test within the past year; 3 others a test 1 to 2 years ago) suggest that this estimate may be conservative.

We acknowledge several limitations of this study. First, while refusal rates in the community cohort were low, and multiple contact attempts were made at all eligible households, including on Saturdays and Sundays, it is possible that non-availability may have biased the community sample toward those with no or less stable income sources; it is not clear whether such biases were amplified or reduced in the MVCT cohort. It is also possible that some MVCT participants may have used different identities, thus precluding their linkage to prior HAARS data. The weighting procedures assumed that testers and non-testers were representative of all testers and non-testers in the four study villages, however, the validity of this assumption cannot be evaluated. Second, risk estimates were based on self reports and thus are likely to represent low estimates; it is also not clear whether such biases apply equally to both cohorts. Third, due to the small number of newly diagnosed HIV infections it was not possible to validate the risk model within our study population. Trend tests, however, suggest an elevated predicted risk among both newly diagnosed female MVCT clients (p = 0.007) and newly diagnosed male MVCT clients (p = 0.043; Table S1). Finally, the study was conducted in four villages in one region of Tanzania, and differences between villages were observed across all domains of investigation, including village characteristics, risk of seropositivity, rates of never testing, and the distribution of correlates of and rates of MVCT participation. This study was not designed or powered to provide estimates at the village level. Future studies should assess the extent to which the observed relationships hold in other areas and whether correlates of MVCT participation differ across villages.

In summary, MVCT successfully attracted men and women with increased exposure risk and appeared to offset economic and logistical barriers to testing, but did not overcome HIV-related stigma or fears of testing or test disclosure. MVCT campaigns and other expanded testing options should be honed further to reduce stigma and attract additional high-risk testers. While optimal testing strategies will differ for different communities, free MVCT campaigns in rural villages appear to be broadly acceptable to wide cross- sections of the intended target population.

## Supporting Information

Table S1 **Correlates of HIV infection from cohort of 5,628 clients presenting for HIV testing between 2005 and 2008 at freestanding HIV voluntary counseling and testing site in Moshi, Tanzania.**
(DOC)

Table S2 **HIV-related stigma assessments and coding rules.**
(DOC)
