# Supplementary material for: Who Tests, Who Doesn't, and Why? Uptake of Mobile HIV Counseling and Testing in the Kilimanjaro Region of Tanzania
Source: PLoS One. 2011 Jan 31;6(1):e16488. doi: 10.1371/journal.pone.0016488 (PMC3031571; doi:10.1371/journal.pone.0016488)
Supplement: Table S1 — Correlates of HIV infection from cohort of 5,628 clients presenting for HIV testing between 2005 and 2008 at freestanding HIV voluntary counseling and testing site in Moshi, Tanzania. (DOC) [file pone.0016488.s001.doc]

|  |  | **Females (N=3,153)** | | | | |  | **Males (N=2,475)** | | | | |
| --- | --- | --- | --- | --- | --- | --- | --- | --- | --- | --- | --- | --- |
|  |  |  |  |  |  |  |  |  |  |  |  |  |
|  |  | Mean (%) | HIV+ (%) | Correlates of HIV | | |  | Mean (%) | HIV+ (%) | Correlates of HIV | | |
|  |  |  |  | OR |  | 95% CI |  |  |  | OR |  | 95% CI |
|  |  |  |  |  |  |  |  |  |  |  |  |  |
| HIV Prevalence |  |  | 18.3 |  |  |  |  |  | 7.1 |  |  |  |
|  |  |  |  |  |  |  |  |  |  |  |  |  |
| Demographic risk characteristics | |  |  |  |  |  |  |  |  |  |  |  |
| Age | 18-24 | 34.4 | 7.1 | *ref* |  |  |  | 31.2 | 1.7 | *ref* |  |  |
|  | 25-29 | 19.8 | 17.6 | 2.97 | *** | [2.08; 4.23] |  | 26.3 | 3.4 | 2.13 | *** | [1.02; 4.44] |
|  | 30-39 | 28.0 | 27.8 | 4.04 | *** | [2.88; 5.67] |  | 30.0 | 12.0 | 8.02 | *** | [4.08; 15.79] |
|  | 40+ | 17.8 | 25.9 | 3.19 | *** | [2.15; 4.73] |  | 12.6 | 16.7 | 11.15 | *** | [5.12; 24.27] |
| Marital Status | Married | 20.8 | 16.1 | *ref* |  |  |  | 20.1 | 9.9 | *ref* |  |  |
|  | Divorced | 13.5 | 25.0 | 1.61 | *** | [1.11; 2.33] |  | 7.1 | 17.0 | 1.82 | *** | [1.04; 3.20] |
|  | Widowed | 11.6 | 41.3 | 3.40 | *** | [2.34; 4.95] |  | 2.2 | 32.7 | 5.23 | *** | [2.29; 11.93] |
|  | Single | 54.1 | 12.6 | 1.07 |  | [0.78; 1.47] |  | 70.6 | 4.5 | 1.29 |  | [0.80; 2.07] |
| Any children |  | 36.9 | 23.1 | 1.42 | *** | [1.03; 1.95] |  | 23.6 | 13.9 | 1.71 | *** | [1.03; 2.84] |
| Unemployed |  | 7.1 | 28.0 | 1.80 | *** | [1.22; 2.66] |  | 2.8 | 14.5 | 2.12 | ** | [0.94; 4.75] |
|  |  |  |  |  |  |  |  |  |  |  |  |  |
| Exposure risk characteristics | |  |  |  |  |  |  |  |  |  |  |  |
| Lifetime partners | 0 or 1 | 40.9 | 9.4 | *ref* |  |  |  | 19.6 | 2.5 | *ref* |  |  |
|  | 2 | 27.1 | 21.0 | 2.34 | *** | [1.74; 3.16] |  | 16.7 | 4.9 | 1.05 |  | [0.47; 2.36] |
|  | 3 | 16.2 | 23.0 | 2.58 | *** | [1.84; 3.60] |  | 14.8 | 6.1 | 0.85 |  | [0.38; 1.92] |
|  | 4-5 | 11.2 | 29.9 | 3.90 | *** | [2.71; 5.62] |  | 21.2 | 6.9 | 1.23 |  | [0.58; 2.61] |
|  | 6 or more | 4.6 | 37.2 | 4.91 | *** | [2.99; 8.06] |  | 27.7 | 12.4 | 1.74 | * | [0.85; 3.53] |
| Any partner tested positive | | 2.3 | 41.9 | 2.04 | *** | [1.02; 4.07] |  | 2.3 | 31.6 | 2.93 | *** | [1.31; 6.54] |
| Suspects any partner has HIV | | 10.6 | 42.0 | 2.24 | *** | [1.59; 3.14] |  | 11.2 | 18.1 | 2.77 | *** | [1.72; 4.46] |
|  |  |  |  |  |  |  |  |  |  |  |  |  |
| Testing history |  |  |  |  |  |  |  |  |  |  |  |  |
| Never tested |  | 54.2 | 31.7 | *ref* |  |  |  | 51.3 | 12.5 | *ref* |  |  |
| Past year |  | 27.1 | 0.9 | 0.01 | *** | [0.01; 0.03] |  | 28.1 | 0.7 | 0.03 | *** | [0.01; 0.07] |
| 1-2 years ago |  | 2.5 | 3.8 | 0.07 | *** | [0.02; 0.23] |  | 3.2 | 2.5 | 0.12 | *** | [0.03; 0.54] |
| >2 years ago |  | 2.4 | 11.7 | 0.25 | *** | [0.11; 0.53] |  | 3.4 | 6.0 | 0.38 | *** | [0.14; 0.99] |
| Unknown |  | 13.8 | 3.7 | 0.05 | *** | [0.03; 0.08] |  | 14.0 | 1.4 | 0.06 | *** | [0.02; 0.16] |
|  |  |  |  |  |  |  |  |  |  |  |  |  |
| Year of test |  | 2006 |  | 0.55 | *** | [0.47; 0.66] |  | 2006 |  | 0.66 | *** | [0.51; 0.86] |
|  |  |  |  |  |  |  |  |  |  |  |  |  |
| Hosmer-Lemeshow goodness of fit | | |  | p=0.4385 | |  |  |  |  | p=0.6297 | |  |
| Area under the ROC curve | |  |  | 0.8866 | |  |  |  |  | 0.8847 | |  |
|  |  |  |  |  |  |  |  |  |  |  |  |  |
| Notes: Estimates from logistic regression models predicting HIV seropositivity. *ref* indicates reference group. | | | | | | | | | | | | |
| Parameter estimates did not differ between rural and urban areas (generalized Hausman tests; males p=0.2273; females p=0.4721). Parameter estimates from gender-specific multivariable logistic regression models were used to calculate three risk indices describing sociodemographic risk, exposure risk, and total risk, respectively. Indices were defined as Ŷ=xβ, with x describing the respective vector of risk indicators, and β describing the corresponding vector of parameter estimates. To allow for a more intuitive interpretation, all indices (sociodemographic risk, exposure risk, total risk) were rescaled to range from 0 to 10, representing the gender-specific minimum and maximum values, respectively, observed in the combined MVCT and community cohorts. Nonparametric trend tests suggest higher predicted risk among 17 newly diagnosed female MVCT clients (p=0.007) and 13 newly diagnosed male clients (p=0.043), relative to MVCT clients who tested negative. | | | | | | | | | | | | |
|  | | | | | | | | | | | | |
|  | | | | | | | | | | | | |
