# Supplementary material for: Who Tests, Who Doesn't, and Why? Uptake of Mobile HIV Counseling and Testing in the Kilimanjaro Region of Tanzania
Source: PLoS One. 2011 Jan 31;6(1):e16488. doi: 10.1371/journal.pone.0016488 (PMC3031571; doi:10.1371/journal.pone.0016488)
Supplement: Table S2 — HIV-related stigma assessments and coding rules. (DOC) [file pone.0016488.s002.doc]

|  |  |  |  |  |  |  | % endorsing |  |
| --- | --- | --- | --- | --- | --- | --- | --- | --- |
|  |  |  |  |  |  |  |  | Coding rule |
| Internal stigma (range 0-6) | | |  |  |  |  |  |  |
| HIV/AIDS is punishment for bad behavior. | | | | |  |  | 57.5 | Agree = 1 |
| People with HIV/AIDS are promiscuous. | | | | |  |  | 50.0 | Agree = 1 |
| I would be ashamed if I were HIV infected. | | | | |  |  | 21.3 | Agree = 1 |
| I would be ashamed if a member of my family were HIV infected. | | | | | | | 18.1 | Agree = 1 |
| In a market, would you buy food from a PLHA if they were not visibly sick?^1^ | | | | | | | 83.7 | No = 1 |
| In a market, would you buy food from a PLHA if they were visibly sick?^1^ | | | | | | | 56.8 | No = 1 |
|  |  |  |  |  |  |  |  |  |
|  | Mean score (sd): | 2.06 (1.56) | |  | Cronbach's alpha: | 0.610 |  |  |
|  |  |  |  |  |  |  |  |  |
| Witnessed stigma (range 0-5) | | |  |  |  |  |  |  |
| Do you know someone who has had the following happen to him/her because of HIV infection? | | | | | | | | |
| Lost customers to buy his/her produce/goods or lost a job. | | | | | |  | 15.5 | Yes = 1 |
| Abandoned by spouse/partner or family. | | | | |  |  | 17.4 | Yes = 1 |
| Teased or sworn at. | | |  |  |  |  | 22.8 | Yes = 1 |
| Physically abused.^2^ | | |  |  |  |  | 6.2 | Yes = 1 |
| Lost respect/standing within the family and/or community. | | | | | |  | 28.9 | Yes = 1 |
|  |  |  |  |  |  |  |  |  |
|  | Mean score (sd): | 0.91 (1.37) | |  | Cronbach's alpha: | 0.774 |  |  |
|  |  |  |  |  |  |  |  |  |
| Source: Nyblade L., MacQuarrie, K. (2006) | | | |  |  |  |  |  |
| ^1^ PLHA = person living with HIV/AIDS | | | |  |  |  |  |  |
| ^2^ Physical abuse added to recommended indicators. | | | | |  |  |  |  |
